# Are Women in Rural India Really Consuming a Less Diverse Diet?

**DOI:** 10.1177/0379572120943780

**Published:** 2020-08-03

**Authors:** Soumya Gupta, Naveen Sunder, Prabhu L. Pingali

**Affiliations:** 1 Tata- Cornell Institute for Agriculture and Nutrition, 5922 Cornell University, Ithaca, NY, USA; 2 Department of Economics, 8243 Bentley University, Waltham, MA, USA

**Keywords:** diet diversity, women, India, food intake, food policy, maternal and child nutrition

## Abstract

**Background:**

It is widely considered that women have less diverse diets than other household members. However, it has been challenging to establish this empirically since women’s diet diversity is measured differently from that of other household members.

**Objective:**

In this article, we compare women’s dietary diversity with that of their respective households and thereby generate a measure of “dietary gap.”

**Methods:**

We measure women’s “dietary gap” by using the difference of homogenized household and woman dietary scores (using the same scales). This is done using primary data on 3600 households from 4 districts in India. Additionally, we show the robustness of our results to variations in scale and recall periods used to construct the diet diversity scores.

**Results:**

Mean difference tests indicate that women consistently consume 0.1 to 0.5 fewer food groups relative to other household members, with the results being statistically significant at the 1% level. The food groups driving this dietary gap are nonstaples like Vitamin A-rich fruits and vegetables, meat/fish/poultry, and dairy.

**Conclusions:**

Results point toward the discrimination faced by women in the variety of the food consumed, the importance of considering comparability in creating indices of diet diversity, and the need to collect more detailed information on diets. To our knowledge, this is one of the first studies to examine dietary discrimination faced by women using common scales.

## Introduction

The intake of a diverse set of foods has been identified as an immediate cause of improved nutritional outcomes in women and children.^1^ One of the most common indicators used to assess the diversity of diets is the Dietary Diversity Score (DDS), which is typically computed as a count of the number of food groups consumed by an individual or household over a given reference period. In this article, we probe differences in diversity in the diets consumed by women and other family members in rural India, for which we use primary data collected from 3600 households across 3 states in India. In doing so we empirically test the commonly held belief that women’s diets in India are less diverse (and potentially less nutritious) as compared to the diet of other household members. Also, in the process, we address issues related to the measurement of DDS and how they can be used for comparisons.

In this analysis, we restrict our attention to 2 types of DDS measures that are predominant in the literature: the Minimum Dietary Diversity–Women (MDDW) and the Household Dietary Diversity Score (HDDS). Minimum dietary diversity–women is based on the number of food groups consumed in the past 24 hours out of a total of 10 food groups,^2^ while the HDDS is based on 11 food groups.^3^ The food groups involved in these calculations are listed in Table 1. Leroy et al^4^ provide a summary of the motivation (and intended uses) behind the creation of these different sets of food group classifications. In general, DDSs have been conceptualized as indicators of food access.^4-6^ While the MDDW has been validated to reflect nutrient adequacy (which reflects diet quality) in women of reproductive age-group, the HDDS has been validated as an indicator for a household’s economic access to dietary energy/higher kilocalories.^4^ In contrast, Coates^5^ suggests that both can be used as measures of nutrient adequacy/quality, while Leroy et al^4^ highlight the traditional distinction between the HDDS and MDDW with respective to diet quality. The MDDW in its original form is meant to be constructed as a binary variable. We however use the 10 food groups to construct a continuous score. To make this distinction between the binary and continuous score clearer, we refer to the latter as the Women’s Dietary Diversity Score (note 1) (WDDS) in the rest of the article.

**Table 1.** Food Groups Included in Dietary Diversity Scores for Women and Households.

Food groups for Minimum Dietary Diversity for Women	Food groups for Household Diet Diversity Score
1. Grains, white roots and tubers, and plantains	1. Cereals
2. Pulses (beans, peas, and lentils)	2. Root and tubers
3. Nuts and seeds	3. Pulses/legumes/nuts
4. Dairy	4. Milk and milk products
5. Meat, poultry, and fish	5. Meat^a^
6. Eggs	6. Fish/seafood^a^
7. Dark green leafy vegetables	7. Eggs
8. Other vitamin A-rich fruits and vegetables	8. Vegetables
9. Other vegetables	9. Fruits
10. Other fruits	10. Oil/fats
	11. Sweets
	12. Spices/ beverages

^a^ Combined together for the purpose of this study.

A key issue in comparing diets of women with other household members is that diet diversity for these 2 groups are measured based on different sets of food group classifications. The household diversity (HDDS) is measured on a scale containing 11 food groups, whereas WDDS is measured based on 10 food groups. In their original form, any differences in DDS between women and households could represent—(1) differences in the scales of the 2 measures, and/or (2) actual differences in the dietary intake of women viz-a-viz their households. This makes it challenging to directly compare the two to make meaningful inferences about gender differences in diets within the household. To overcome this issue, we homogenize the scale(s) used to compute the DDSs for women and households in order to be able to draw inferences about gender disparities in diets. Using the same scale for both the groups involves constructing both the women’s and household’s DDSs using either the WDDS definition (10 food groups) and/or using the HDDS definition (11 food groups), and making comparisons across them within the same definition.

Although an uncommon practice in the literature, this approach has been adopted in a few recent studies. Koppmair et al^7^ use the 12 food groups of the HDDS to construct DDSs not just for the household but also for women and children in Malawi. They find a significant association between these DDS and both, the number of food groups produced as well as household market access. Jones^8^ computes the HDDS based on the 10 food groups specified in the MDDW and finds a significant association between on-farm species richness and household dietary diversity in Malawi. However, to the best of our knowledge, we are not aware of any study that homogenizes the scales for DDS calculation for 2 groups (women and households) in order to analyze dietary gaps between them. It is important to note that in our use of the common scales we make no assumptions about the efficacy of the 10-point scale in reflecting the dietary quality at the household level (as in Jones^8^) nor do we claim that the 12-point scale reflects dietary quality of women (as in Koppmair et al^7^). We also do not compare the results from the 2 scales to ascertain which one “better reflects dietary diversity,” something that has been flagged as an issue.^9^ In this study, the homogenous scales are simply created to compare the dietary diversity of women with that of their other household members.

Our results indicate that on average women consume 0.1 to 0.5 fewer food groups as compared to other household members. These differences are primarily focused in more nutritious food groups (like pulses, green-leafy vegetables, fruits, and dairy). We also find that the results are similar across the 3 states (Uttar Pradesh, Bihar, and Orissa) in our sample, thus pointing toward a “universality” in this *stylized fact*. Our findings are robust to changes in the statistical procedure used, variations in the scale of the diet diversity measure and to altering the recall period used in constructing DDS. Additionally, we note that since our analysis focuses solely on the difference in diet diversity at the extensive margin (ie, differences in food groups consumed), our results are potentially a lower bound of the discrimination faced by Indian women. Differences in the intensive margin of food consumption (quantities of different foods that are consumed) are beyond the scope of this analysis, and represent a potential avenue of future research.

Our study makes the following contributions to the literature. First, we provide a detailed assessment of diet diversity among women and households in rural India. This part of the analysis contributes to the scant quantitative evidence on diet diversity in India (as compared to other geographies)—which is evidenced by the fact that only 3 out of 45 recent meta-analyses on dietary diversity were based on India.^10^ This is especially critical given the high rates of undernutrition in India, especially among women—nearly 55% women in rural India are anemic and another 30% are underweight as characterized by low body mass index.^11^ Second, we collect (and use) primary data on dietary consumption for both, women and households separately, which allows for accurate inference for both of these groups. Very few studies have computed DDS for both women and household using the same data set, especially in the Indian context (with the only exception being Padmaja et al^12^)Third, to our knowledge this is the first study to use detailed primary data to provide empirical evidence of the dietary gap faced by Indian women (especially in rural areas). Its importance is magnified in a context like India, due to the critical role of women in cooking and feeding practices.^13-16^ Additionally, by studying the disadvantages that Indian women face with respect to diet diversity, we add to the documented evidence of similar challenges faced by them in other socioeconomic outcomes.^17-20^ Fourth, we develop and implement a novel measure of dietary gap between women and other household members, which makes a methodological contribution on the use of DDSs as a measure for “differences” in diet diversity of 2 distinct respondent groups.

## Methods

### Sample and Data Collection

This article uses primary data that was collected as part of a baseline survey designed and implemented by the program on Technical Assistance and Research for Indian Nutrition and Agriculture (TARINA) in India. The TARINA program, led by the Tata-Cornell Institute of Agriculture and Nutrition at Cornell University, is a consortium of research organizations and field-level development organizations that are working toward the design, promotion, and evaluation of nutrition-sensitive food interventions in 4 districts of India: Munger (Bihar), Maharajganj (Uttar Pradesh or UP), Kalahandi (Odisha), and Kandhamal (Odisha). The TARINA Baseline Survey was implemented across a total of 3600 households in 120 villages. A 2-step sampling strategy was followed. In the first step, based on population size and the geographical areas in which the partner organizations operated, 30 villages were selected in each district. In the second step, 30 households per village were selected randomly. This random selection of households was done after a census of all households was conducted within each of the selected villages (from step one). Within each household an index man and index woman were identified as the main respondents for the survey.

The survey was divided into 2 parts, depending on the type of information being collected and the person who would be most likely to provide accurate responses. In the first part, the index male was asked questions related to household demographics, socioeconomic status, agricultural cropping practices, land use, and livestock ownership. The second part was administered to the index woman who was asked questions related to food access, Infant and Young Child Feeding practices, Water Sanitation and Hygiene, group membership, and empowerment in agriculture. The women were also administered a module on individual and household-level dietary intake over the past 24 hours and frequency of food intake over the past 7 days. In addition to the household and individual-level data, the TARINA baseline survey also collected a host of village-level indicators on demography; presence and access to government services like health, education, Public Distribution System; and market-access indices like distance and time to market. Data collection took place between January 2017 and May 2017. The study was approved by the Institutional Review Board at Cornell University. Informed consent was obtained from each respondent and recorded electronically.

### Construction of Women’s Dietary Gap

For this study, data on dietary intake in the past 24 hours (and 7 days) was collected at the individual (woman) and household level using a list of foods that are commonly consumed in the districts within our sample. This list was created based on information that the study team gathered from secondary sources, and via a pilot study (pretesting) and focus group discussion with community members and key stakeholders in the program locations. Here, care was taken so as to make the list as exhaustive as possible, in order to capture all food items related to the food groups that are included in the WDDS and HDDS definitions of dietary diversity. This process led to a final tally of 37 food items. For each of these 37 food items, the survey recorded information about whether or not it was consumed by (a) the index woman (note 2) and (b) by any other member of the household in the past 24 hours (7 days). The WDDS and HDDS was calculated as the sum of the food groups consumed (Table 1) in the past 24 hours by the woman and household, respectively.

Typically, the 10-point WDDS would be calculated for the women and the 11-point HDDS for the households. A simple measure of dietary differences would be to look at their difference. However, since the underlying scales are different it will not be meaningful to interpret this difference. Therefore, we homogenize the scales used to compute the DDS for women and households. To do this, we compute the DDS for women a second time, based on the 11 food groups in the HDDS. Similarly, we also compute the HDDS using the 10 food groups included in the WDDS. This way we have a set of DDSs—for both women and households—based on the 10 food groups as well as the 11 food groups. This then enables us to compute the dietary gap using each of the 2 scales: DietaryGap_10_ (in Equation 1) and DietaryGap_11_ (in Equation 2) refer to the difference between the woman’s and household’s DDSs based on the 10-point and 11-point scale respectively (note 3).

$$DietaryGap_{10}=MDDW_{10}-HDDS_{10}\;\left(1\right)$$

$$DietaryGap_{11}=MDDW_{11}-HDDS_{11}\;\left(2\right)$$

By homogenizing the scale, any difference between the 2 scores can be attributed to an objective difference in dietary intakes of the 2 groups, and not to differences in the scales at which they are measured. A difference less than zero implies that women consume fewer food groups than the rest of the household. This occurs in 23% of the households—that is, in almost a quarter of the households the woman’s DDS is less than that of the household (by at least one food group). We further examine if these differences are concentrated in particular food groups or not (note 4).

### Empirical Methodology

Our objective is to empirically examine whether there exists a gap in diet diversity between women and other household members in rural Indian households. Equations 1 and 2 refer to the women’s dietary gap based on the 10-point and 11-point scale respectively. In order to probe any gaps in diets between women and other members of the household, we conduct one-sided *t* tests of mean differences between the DDSs for women and households. These are one-sided because the woman’s dietary score cannot be greater than that of the household (when using the same scale). Therefore, we test the null hypothesis of no differences between women and household diets against the alternative hypothesis that women consume fewer food groups than other household members.

Next, we unpack the DDSs and look at how consumption of different food groups varies between women and other household members. We calculate the proportion of households that did or did not consume a given food group in the total sample. For those households that did report having consumed the food group we further bifurcate them into 2 groups—one, where both the household and the woman report having consumed the food (HH-Y, IND-Y) and second, those in which the household consumed the food group but the woman did not (HH-Y, IND-N). It is the latter that brings out the specifics of the food groups that are contributing to the overall gap in dietary diversity of women, relative to their household. We also conduct robustness checks to analyze the sensitivity of our findings.

## Results

### Status of Dietary Diversity Across Districts Using 24-Hour and 7-Day Recall

As per the 10-item definition of individual dietary diversity discussed earlier, the mean DDS for women in our sample is 4.3 food groups. On average, women in the 2 districts in Odisha consume 4.7 and 4.8 food groups (Table 2), which is almost one food group higher than the diet diversity score for women in Munger (Bihar) and Maharajganj (UP). Further, when broken down into specific food groups, we find that a typical diet for women across the 4 districts consists of cereals, pulses, and other vegetables, while the consumption of micronutrient-rich food groups (like green leafy vegetables [GLV], Vitamin A rich fruits and vegetables, meat/fish/poultry, and eggs) is very low (less than 30% in any given district). Nearly 50% of the women in each district have DDSs lower than the mean, suggesting that the overall low diversity of diets is not being driven by a few extreme values. Table 2 also presents results for household-level DDSs. Based on the HDDS definition, the average household in the sample consumes 6 food groups over a given 24-hour period. Additionally, we notice that there are no significant regional differences in the HDDS scores with the 24-hour recall. We find similar patterns in the results using the 7-day recall period. Mean DDS from Table 2 suggest that women are consuming fewer food groups as compared to their households. However, given that the 2 indices are measured on different scales, the difference does not have a meaningful interpretation.

**Table 2.** Average Diet Diversity Scores and Proportion of Women With Dietary Diversity Scores Below Mean (24-hour and 7-day Recall).

	24-Hour recall				7-Day recall			
	WDDS^a^		HDDS^a^		WDDS^a^		HDDS^a^	
District	Mean	Below mean (%)	Mean	Below mean (%)	Mean	Below mean (%)	Mean	Below mean (%)
Munger	3.8	40.8	6.0	34	5.4	55	7.2	50.7
Maharajganj	3.8	48.4	5.8	44	5.6	48.8	7.2	54.5
Kandhamal	4.8	51.7	6.0	40	7.1	53.6	8	35
Kalahandi	4.7	49.7	6.0	37	6.9	40.1	7.8	40.1
Full Sample	4.3	61.5	6.0	38.7	6.2	54.2	7.6	47.3

Abbreviations: HDDS, Household Dietary Diversity Score; MDDW, Minimum Dietary Diversity–Women; WDDS, Women’s Dietary Diversity Score.

^a^ WDDS is the Women’s Dietary Diversity Score on a scale of 0-10. HDDS is the Household Dietary Diversity Score on a scale of 0-11. The districts are in the following states: Munger (Bihar), Maharajganj (Uttar Pradesh), and Kandhamal & Kalahandi (Odisha). Difference refers to the shortfall in MDDW relative to the HDDS in each district.

These findings are consistent with a bulk of the literature on diet diversity in India. Using data from the India Human Development Survey, Bhagowalia et al^21^ find that Indian households on average consumed 6 (out of 13) food groups in the previous 30 days. Kavitha et al^22^ use data on 3 districts in Telangana and Maharashtra, from the Village Dynamics in South Asia program, and find that the average HDDS ranges between 7 and 9 food groups, out of a total of 12 food groups. Using a different primary data source, Shashikantha et al^23^ also find that most women in their sample in Karnataka consumed 5 of 9 food groups, with about 30% of them consuming fewer food groups. In other parts of South Asia DDSs have been as high as 12 out of 13 food groups for households in Nepal^24^ (Pellegrini and Tasciotti) and as low as 4 out of 9 food groups for women in Bangladesh.^25^

### Women’s Dietary Gap Across Recall Periods

Table 3 summarizes the dietary diversity gap for both the 10- and 11-point scales based on 2 different recall periods (24-hours and 7 days). Comparisons of the DDS using the same scale suggest that the average woman is consuming significantly fewer food groups than other household members. The size of the difference varies depending on the scale and the recall period, but all the differences are statistically significant at the 1% level. This points toward a persistent shortfall in women’s diets, when compared to the rest of the household.

**Table 3.** T-test of Differences Between Women DD and Household DD.

	#Food groups^a^	24-Hour recall			7-Day recall		
		Women’s Dietary Diversity Score	Household Dietary Diversity Score	Women’s dietary gap^b^	Women’s Dietary Diversity Score	Household Dietary Diversity Score	Women’s dietary gap
Full sample	10	4.28	4.49	−0.21^c^	6.23	6.33	−0.10^c^
	11	5.58	5.98	−0.39^c^	7.04	7.56	−0.52^c^
Munger	10	3.86	4.06	−0.20^c^	5.38	5.46	−0.08^c^
	11	5.56	6.04	−0.48^c^	6.60	7.16	−0.56^c^
Maharajganj	10	3.75	4.07	−0.32^c^	5.55	5.65	−0.10^c^
	11	5.28	5.79	−0.51^c^	6.69	7.22	−0.53^c^
Kandhamal	10	4.76	4.97	−0.21^c^	7.10	7.21	−0.11^c^
	11	5.72	6.04	−0.32^c^	7.50	7.96	−0.46^c^
Kalahandi	10	4.72	4.87	−0.15^c^	6.89	6.99	−0.10^c^
	11	5.76	6.04	−0.28^c^	7.37	7.82	−0.45^c^

Abbreviation: WDDS, Women’s Dietary Diversity Score.

^a^ The number of food groups refer to the scale of the score. The 10 food groups correspond to the WDDS while the 11 food groups correspond to the HDDS scale.

^b^ Women’s dietary gap = Women’s Dietary Diversity Score − Household Dietary Diversity Score.

^c^Significance at 0.1% level (p<0.001)

**Figure 1.**
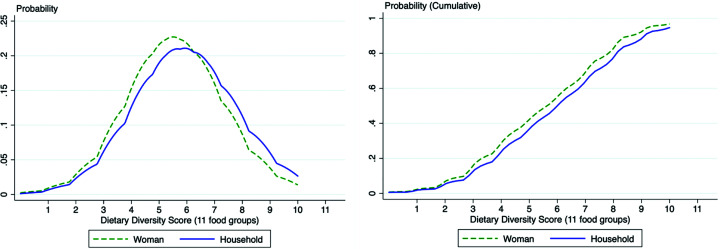
Distribution of Dietary Diversity Scores for women and household.

A comparison of the distribution of DDSs for the woman and the household, both based on the 11-point scale, is provided in Figure 1. The figure on the left shows that the women’s score has a higher probability in the lower part of the score distribution, whereas the household score has higher probability in the higher diet diversity scores. A similar pattern is observed in the cumulative probability—the distribution for the woman’s score is always to the left of the household score. This implies that the plot of women’s diet diversity first order stochastically dominates the one for the household (women have more probability density in the lower part). Put together, these illustrate the fact that overall women have lower diversity scores than households.

**Figure 2.**
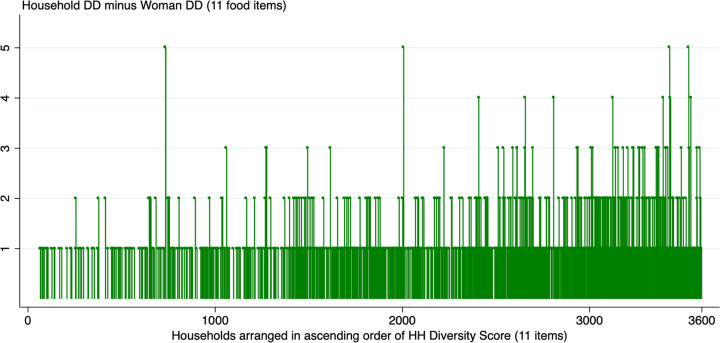
Difference in Dietary Diversity Score of household and the woman, 11-point scale.

Figure 2 shows a plot of the differences in DDSs for women and their households based on the 11-point scale. This graph plots the difference between household and women diet diversity scores (y-axis). Here, the households are arranged in ascending order of household diet diversity (x- axis). The size of the line gives the value of the difference between the women’s and their respective household’s DDS, while the lack of a line for a particular household (white spaces) imply no differences between the consumption reported for the household and the woman. This graph shows that women consume less than the household across the whole distribution, and that the differences are not restricted to either low or high values of household diversity. This implies that differences in diets are present in households of all types, and are not restricted to certain kinds of households.

**Figure 3.**
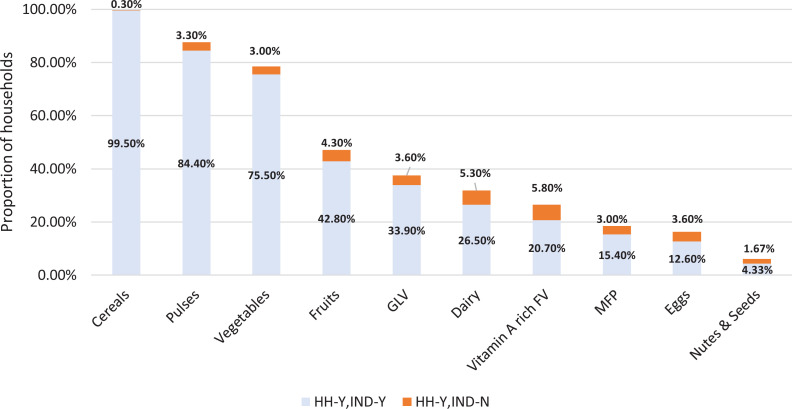
Proportion of households that reported consuming each food group, by woman’s consumption status (24-hour recall), 10-point scale. Note: The total length of each bar represents the percentage of households that consumed the particular food group. It is divided into 2 categories—the blue region represents cases where the woman also reported consuming items in the food group, while the orange region represents cases where the woman did not consume that food group. Please note that the remaining percentage of households (HH) did not consume the food group.

Next, we analyze the specific food groups in which women are particularly lacking. This is critical in examining the potential impact that this dietary shortfall can have. In Figure 3 we provide food-group level information on consumption by households and women. For each food group 2 stacked bars are presented. The first (lower) bar reports the percent of households where both women and other members consumed a given food group. The second (upper) bar reports the percent of households where the food group was consumed by other members but not by the woman. It can be seen that while the overall consumption of staple cereals and pulses is high, it is the perishable, micronutrient-rich nonstaple food groups that are being consumed to a lower degree. This becomes important because not only are smaller proportions of households consuming these food groups, but within the households that consumes these food categories, not all women consume these foods. For instance, GLV (37.5%), Vitamin A rich fruits and vegetables (26.5%), meat-fish-poultry or MFP (18.4%), and dairy products (31.8%) are less commonly consumed but the gender differences are high—9.6% GLV, 22% Vitamin A rich fruits and vegetables, 16% MFP, and 16.7% dairy—of these have women who do not report consuming it.

## Robustness Checks

We check the sensitivity of the results to changing the recall period used for assessing the dietary diversity gap. The recall period is an important component of the computation of diet diversity scores. Survey respondents are asked for their consumption of different food items for a predetermined recall period, where typical recall periods are 24-hours (example—WDDS, HDDS), 3-days,^26^ 7-days (note 5).^27-30^ The recall period is especially relevant for less frequently consumed foods,^31^ which are most often consumed outside the house (note 6).^32^ This difference would in all probability be higher in contexts where people consume a wider variety of foods or they consume many food items at a low frequency (where shorter recalls might not capture this diversity). In addition, Savy et al^26^ in their comparison of different recall periods point to the importance of accounting for atypical days like market days in the measurement of dietary diversity, as do Pingali and Ricketts (note 7).^33^ To account for these factors and to show the robustness of our results to changes in recall period, our study uses 2 different recall periods—24 hours and 7 days. Results in Table 3 suggest that the findings remain consistent across the 2 reference periods, thus allaying concerns that our results are driven by the recall period of the Dietary Diversity Score.

In another robustness check we analyze how our results change when we alter the scale of the DDSs. We compute the DDSs for women and households (and therefore the dietary diversity gap) twice—once using the 10 WDDS (women) food groups and then using the 11 HDDS (household) food groups. We verify if upon changing the scales, the evidence on dietary diversity gap remains the same or not. If the 2 scales provide similar results, then it points toward a case for the use of either scale in such an analysis. If the results are not the same, then it implies the need to exercise caution in the choice of scale adopted for such comparisons.

We show that the results remain robust to the use of Welch *t* test. The Welch test is supposed to be more appropriate in the case that the 2 populations being compared have unequal variances or sample sizes. In our case, the sample sizes are same (3600 observations), but the variances of the samples could be different. Applying the changed formula, the *P* values of the differences fall but are all significant at the 1%level (results available on request).

If we compare the gap *within* each definition, across recall periods, we find that the women’s dietary gap actually reduces with a longer recall period. The dietary gap based on the 10-point scale becomes half in size when we look at consumption patterns over 7-days as compared to a 24-hour recall. Similarly, the dietary gap reduces by 25% when the 11-point scale is used. In appendix A we discuss how the results vary *across* definitions of the dietary diversity scores.

## Conclusion

In this article, we use primary data from 3600 households in India to test the existence of a gap in the dietary diversity of women relative to other household members. Based on conventional measures of dietary diversity, we find that women on an average consumed 4 food groups in the previous 24-hours, while other household members consumed almost 6 food groups. The challenge of directly comparing these numbers is that they are measured on 2 different scales. We overcome this by calculating the DDS for women and households on the same scales and then use it to make comparisons. Such comparisons are useful in as much as they allow us to leverage the advantages of DDSs (like ease of design, administration, and analysis) in estimating whether or not there is parity in the dietary intake across different household members. Our methodology of homogenizing the scales of DDSs allows us to contribute to the policy discussion on dietary diversity.

We find strong evidence that women consume less diverse diets than their households, and these findings are consistent across different study sites and definitions of diet diversity. Our findings on the existence of a dietary gap faced by women are in line with existing empirical evidence from different parts of the world that show a bias against women in intrahousehold food allocation (Hadley et al,^34^ Gittelsohn,^35^ Harris-Fry et al^36^). Many factors have been identified to be associated with these gender differences in food allocation. Harris-Fry et al^36^ (2018) stresses the importance of income, bargaining power, social status, tastes/preferences, and interpersonal relationships in determining food allocations within a household. Similarly, lopsided intrahousehold food allocation has also been associated with the role of different family systems (Madjdian and Bras) and women eating after all other members have eaten (Gittelsohn^35^). Additionally, we find that women’s diets are particularly lacking in nonstaple food groups like Vitamin A rich fruits and vegetables, dairy, eggs, and GLV. Shashikantha et al^23^ too find that while all women consumed starchy staples, 85% consumed pulses and less than 5% consumed egg and meat products in their sample from Karnataka, India.

A disaggregated analysis of the scores can further shed light on the specific food groups that are contributing to this gap. This can help define policy priorities on which food groups need to be targeted for particular subpopulations. Also, it indicates the need for a “gendered lens” in nutrition policy formation. Targeting of programs toward key demographics might bring larger “bang for the buck” and help address historical disadvantages. Additionally, benefits accruing to women of child-bearing age may lead to large intergenerational benefits through effects on child health.^37-42^ These further enhance the benefits of these programs.

Methodologically this study is one of the first ones to provide robust documentation of such a gender-gap in diets among Indian households. Previous studies have been unable to explore this gap due to the inability to credibly compare woman and household DDSs within the same context. This is primarily owing to the differences in their scales, and to potential disparities in recall period. Our results indicate that women’s dietary diversity is less than that of their households when the same scale is used. Data limitations prevent us from comparing women’s dietary intake to that of specific members of her household, for example the man or the children. Having said that, when the HDDS is used, questions related to consumption of different food groups are asked across all members of the household (eg, *did a member of your household consume ___ food group in the previous 24 hours?*). So, while we are not able to identify exactly which household member (say, the man or the child) may be consuming a more diversified diet as compared to the woman, we can still make the claim that there is at least one other member of the household who does in fact have a more diversified diet than the woman (note 8).

Another aspect of the use of the same scale for the WDDS and HDDS is related to the need for a renewed conversation around the underlying premise for the different food groups. For instance, as dietary patterns change it may be worthwhile to revisit the food group classification such that it accounts for increased consumption of fat-rich foods by women just as it accounts for the consumption of micronutrient-rich foods by households. Although women’s intake of micronutrient rich foods is pertinent to their health, it may warrant tracking at the household level too, as the consumption of such foods has gradually risen among marginal farmers—especially as the reliance of smallholders on markets as a source of diverse foods has grown.^43^ Analogously the increasing consumption of “largely nutrient-poor”^4^ food groups (oils/ fats, sweets, spices/ beverages) and the associated increasing burdens of overnutrition are well documented.^44^ This make their measurement important not only at the household-level (in HDDS) but also in capturing the energy-rich, nutrient-poor food consumption at the individual-level (women). By not accounting for these food groups, the MDDW is restricted in its scope/ability to capture the entire spectrum of the quality of consumption patterns.

Our work also highlights the need for detailed primary data on dietary intake. The dietary gap measure that we use contributes to the set of metrics that can be used to assess nutritional outcomes for different populations. This is in line with data deficiencies identified by the Global Panel on Agriculture for Food Systems and Nutrition in the areas of diet quality and quantity,^45^ which states “there is a need for data, wherever feasible, to be stratified by subregion, gender, age and socioeconomic status, to more effectively guide policy.” In fact, the Panel suggests collecting data based on the MDDW as a proxy for a household’s dietary quality.^45^ ^(p7)^ In line with that, our body of work can possibly be used to reflect differences in dietary quality between members of the household. Moreover the policy implications of information on women’s dietary gap can address the “lack of critical data on which to base important decisions and design interventions” that characterizes the formulation of policies related to diets.^45^

Having said that, we note that there are still many avenues that need to be explored to further strengthen this body of research. Future research efforts can accommodate for seasonal variations in consumption. Additionally, data on portion sizes could potentially provide more reliable estimates of any differences in dietary intake. This would also enable the identification of key food groups for intervention (eg, from the point of view of iron deficiency, we may be interested in looking at the dietary gap specifically with respect to iron-rich foods like meats and pulses). Moreover, differences can also be analyzed in terms of not just gender but also different age-groups, local caste/community groups, and other dimensions of socioeconomic disadvantage.

## Appendix A

### Comparing Results with Different Scales

The overall significance of the results shows that the dietary differences are agnostic to scale and recall period. In general, we find that the gap based on an 11-point scale is greater than the gap based on the 10-point scale for a given district and given recall period. For the 24-hour recall period, the magnitude of difference based on a 10-point scale is half of that based on an 11-point scale, whereas the ratio in the gaps is around 5 for a 7-day recall period. These differences in the dietary gap *across* scales can largely be attributed to the way food consumption is aggregated to create the Dietary Diversity Scores in each case. In the 10-point scale, the difference between individual and household scores is less, as compared to the 11-point scale. What this means is that, on average, there are fewer households where the household is consuming but the woman isn’t consuming, relative to the 11-point scale. This is reflected in the table below (HH-Y, IND-N, total sample). What we see is that on average 4% to 5% households are there where someone in the household is consuming a food group but the woman isn’t, when we use the 10-point scale. This difference increases when these 10 food groups are expanded to the 11-food groups used in the HDDS. This is because of an interplay of the following 3 factors:

- One is that, certain disaggregated food groups are now clubbed together into 2 broad groups—fruits and vegetables—and the proportions for these are on average 3% gap.
- The second thing that happens is that since cereals and tubers are now separated, the gap increases since nearly 4% households have the HH-Y, IND-N status.
- The third thing that happens is that the 11-point scale includes 3 food groups that are not there in the WDDS food groups at all—sweets, oils/ fats, and spices/beverages. It is here that the proportion of HH-Y, IND-N is very high. It is nearly 16% households for sweets. It is these high differences that are ultimately getting reflected in a greater gap when the 11-point scale is used, relative to the 10-point scale.

Having said that we would like to caution that we cannot compare the absolute value of the difference across the 2 scales. The Dietary Diversity Scores for the woman and household are both computed separately for the 10-point and 11-point scale so that comparisons can be made *within* each definition (and not *across* definitions).

**Table A1.** Proportion of Households That Report Consuming Food Groups Where the Woman Did Not Consume It.

Food group	% Responses with Household-Yes, Woman—No	
	10-point scale	11-point scale
Cereals	0.3%	0.5%
Tubers		3.7%
Pulses	3.3%	3.2%
Nuts and seeds	1.7%	
GLV	3.6%	
Vitamin A rich fruits and vegetables	5.8%	
Other fruits	4.3%	
Other vegetables	3%	
Fruits		4.5%
Vegetables		2.6%
Dairy	5.3%	5.3%
MFP	3.0%	3.0%
Eggs	3.6%	3.6%
Sweets		15.8%
Oils/fats		0.6%
Spices/ beverages		0.6%

Abbreviations: GLV, green leafy vegetables; MFP, meat-fish-poultry.

## Notes

1. Our construction of the WDDS is different from a similar WDDS put forth by the FAO that computes Dietary Diversity Scores for women based on 9 food groups. https://a4nh.cgiar.org/2015/09/22/get-to-know-the-new-indicator-for-measuring-womens-dietary-diversity/

2. Nonpregnant, nonlactating woman of reproductive age (15-49 years) who is also the primary person in charge of food preparation in the household. May or may not be the spouse of the index man.

3. For the purpose of our study, 2 food groups within the HDDS are combined into 1 category: meat and fish/seafood. Therefore, the HDDS score in this analysis ranges between 0 and 11. For both the MDDW and the HDDS, a larger score signifies a higher diversity in the underlying diet.

4. Note that differences in particular food group could arise even if the overall dietary score for the woman and the household are the same.

5. The 30-day recall period has been used in some surveys (example—National Sample Survey in India), but is less popular than the aforementioned ones.

6. There is limited evidence looking at how diversity scores vary with changes in recall period. Prior evidence from Burkina Faso suggests that even though the local diets lack much variety, a 1-day recall is unable to capture the diversity that is shown with a 3-day recall.^26^

7. In our analysis we have also taken care to note that either recall periods should not have contained any atypical days—festivals, market days, and so on.

8. The HDDS is likely to exceed the WDDS because it describes consumption by *everyone* in the household whereas the WDDS describes consumption by *one person* in the household. Therefore, any difference within the same household will almost always favor the HDDS, *even when the same scale is used*.
